# Antioxidant Properties and PC12 Cell Protective Effects of a Novel Curcumin Analogue (2*E*,6*E*)-2,6-Bis(3,5- dimethoxybenzylidene)cyclohexanone (MCH)

**DOI:** 10.3390/ijms15033970

**Published:** 2014-03-05

**Authors:** Gui-Zhen Ao, Xiao-Jing Chu, Yuan-Yuan Ji, Jian-Wen Wang

**Affiliations:** College of Pharmaceutical Sciences, Soochow University, Suzhou 215123, China; E-Mails: aoguizhen@suda.edu.cn (G.-Z.A.); cxj945@163.com (X.-J.C.); yuanyuanji11@gmail.com (Y.-Y.J.)

**Keywords:** curcumin analogue, free radical scavenger, hydrogen peroxide, PC12 cells, apoptosis

## Abstract

The antioxidative properties of a novel curcumin analogue (2*E*,6*E*)-2,6-bis(3,5-dimethoxybenzylidene)cyclohexanone (MCH) were assessed by several *in vitro* models, including superoxide anion, hydroxyl radical and 1,1-diphenyl-2-picrylhydrazyl (DPPH) radical scavenging and PC12 cell protection from H_2_O_2_ damage. MCH displayed superior O_2_^•−^ quenching abilities compared to curcumin and vitamin C. *In vitro* stability of MCH was also improved compared with curcumin. Exposure of PC12 cells to 150 μM H_2_O_2_ caused a decrease of antioxidant enzyme activities, glutathione (GSH) loss, an increase in malondialdehyde (MDA) level, and leakage of lactate dehydrogenase (LDH), cell apoptosis and reduction in cell viability. Pretreatment of the cells with MCH at 0.63–5.00 μM before H_2_O_2_ exposure significantly attenuated those changes in a dose-dependent manner. MCH enhanced cellular expression of transcription factor NF-E2-related factor 2 (Nrf2) at the transcriptional level. Moreover, MCH could mitigate intracellular accumulation of reactive oxygen species (ROS), the loss of mitochondrial membrane potential (MMP), and the increase of cleaved caspase-3 activity induced by H_2_O_2_. These results show that MCH protects PC12 cells from H_2_O_2_ injury by modulating endogenous antioxidant enzymes, scavenging ROS, activating the Nrf2 cytoprotective pathway and prevention of apoptosis.

## 1. Introduction

**Figure 1.**
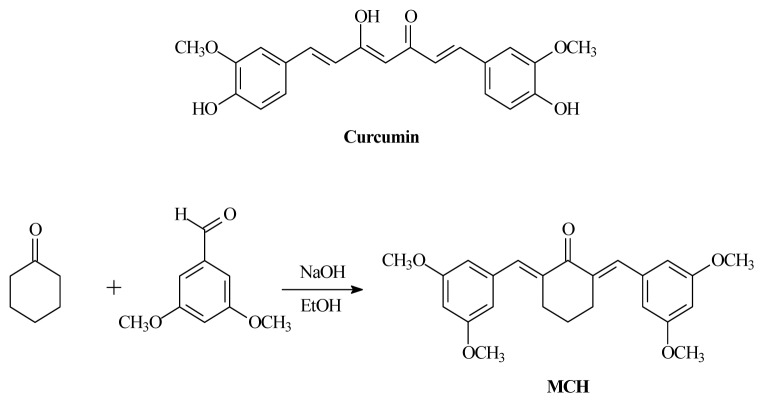
Chemical structure of curcumin and synthesis of (2*E*,6*E*)-2,6-bis(3,5-dimethoxybenzylidene)cyclohexanone (MCH).

Curcumin (diferuloylmethane, Figure 1) is isolated from the rhizome (turmeric) of the herb *Curcuma longa* L. and has been widely used in traditional Indian and Chinese medicine for the treatment of many diseases including inflammation, dyspesia, respiratory disorders, arthritis and others [1]. Curcumin has exhibited diverse pharmacological activities such as anti-carcinogenic, anti-inflammatory, antioxidant and antimicrobial activities [2]. Furthermore, some reports have suggested possible beneficial effects of curcumin on the animal models and human studies of Alzheimer’s disease [3].

However, curcumin is limited in its clinical efficacy owing to its poor absorption across the gut, limited tissue distribution, rapid metabolism, and its subsequent elimination from the body [4]. To circumvent this limitation, several approaches have been carried out, including the design and synthesis of novel curcumin analogues [4–8]. Previous studies have demonstrated that some of those analogues have higher antioxidant activity than curcumin [5,9]. It was believed that the instability of curcumin structure was attributed to the active methylene group and β-diketone moiety [10,11]. Omitting the active methylene group and one carbonyl group leading to 1,4-pentadiene-3-ones, cyclopentanone and cyclohexanone analogues resulted in more stable compounds possessing antioxidative properties [12]. Oxidative stress is involved in the pathogenesis of neurodegenerative disorders such as Parkinson’s and Alzheimer’s diseases, and curcumin analogues have been proven effective in neuroprotection [3,13]. In rat pheochromocytoma PC12 cells, a model of neuronal cells, curcumin, demethoxycurcumin and bisdemethoxycurcumin attenuated neuronal cell death caused by β-amyloid-induced oxidative stress [14]. In light of the above discoveries and in order to highlight the interest of designing neuroprotective agent, a new curcumin analogue, (2*E*,6*E*)-2,6-bis(3,5-dimethoxybenzylidene)cyclohexanone (MCH, structure given in Figure 1), has been developed and is expected to exhibit antioxidant properties and protective effects against H_2_O_2_-induced cytotoxicity in PC12 cells. Further research into the development of the synthetic analogues has resulted in the discovery of several active molecules [15]. In the present study, we evaluated the antioxidant potential of MCH by DPPH/superoxide/hydroxyl radical scavenging, observed its effect on the mitochondrial membrane potential (*ψ*_m_), reactive oxygen species (ROS) generation, glutathione (GSH) levels, catalase (CAT) and superoxide dismutase (SOD) activities, as well as the expressions of cleaved caspase-3 activity in PC12 cells. These studies were carried out to assess the possible protective effects from oxidative stress by MCH on neurons, as a preliminary step in the understanding of its mechanism of action.

## 2. Results

### 2.1. Free Radical Scavenging Activities and Reducing Power of MCH

MCH and curcumin were subjected to scavenging experiments on DPPH, superoxide anion and hydroxyl radicals and the results are shown in Table 1. MCH was found to be a stronger scavenger on superoxide anion, with a lower *EC*_50_ value of 64.78 ± 7.72 μM than curcumin (*EC*_50_ = 88.09 ± 5.97 μM) or the positive control vitamin C (*EC*_50_ = 100.93 ± 4.10 μM). However, MCH exhibited weaker scavenging capacity on DPPH and hydroxyl radical than those of curcumin.

### 2.2. Stability of MCH and Curcumin

**Figure 2.**
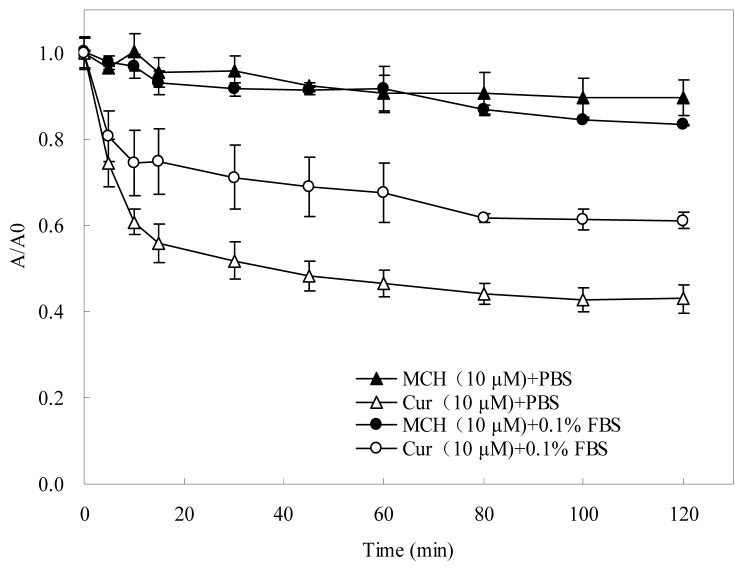
Stability comparison of MCH and curcumin measured by visible absorption in the absence or presence of 0.1% FBS. *A*_0_ means absorption of the solution measured at 410 nm at 0 min; *A* means absorption of the solution measured at 410 nm. Data presented are the means ± SD of results from three independent experiments.

To investigate the stability of MCH and curcumin in physiological media, the absorption variation was measured in phosphate-buffered solutions (pH 7.4) in the presence or absence of 0.1% fetal bovine serum (FBS) under daylight condition (Figure 2). It was found more than 38.8% curcumin degraded very rapidly in the absence of 0.1% FBS within 120 min. In the presence of 0.1% FBS, curcumin was a little stable. In contrast, the absorption of MCH remained almost unchanged, no matter 0.1% FBS was present or absent, indicating that the monoketone-linked curcumin analogue MCH was much more stable during the measurement.

### 2.3. Cytotoxicity of MCH in PC12 Cells

**Figure 3.**
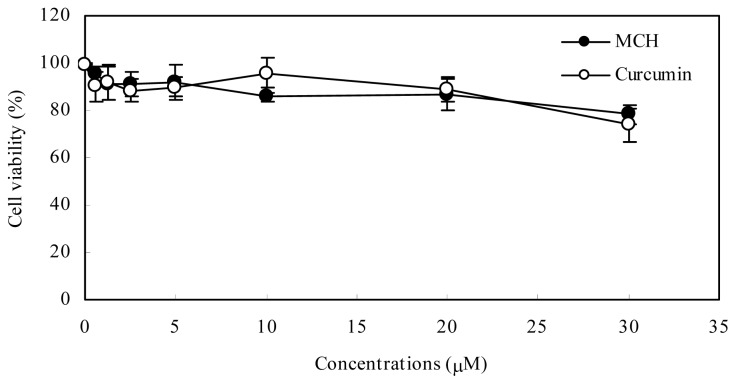
Effects of MCH and curcumin on PC12 cells. Cells were treated with 0.63–30 μM MCH or curcumin for 24 h. Data presented are the means ± SD of results from three independent experiments.

After 24 h treatment with MCH at concentrations between 0.63 and 5 μM, the reduction in cell viability was no greater than 9% (Figure 3). At the highest concentration (30 μM) evaluated, the reduction in cell viability was 21.8%. There is no significant difference in cytotoxicity induced by MCH and curcumin in PC12 cells.

### 2.4. MCH Protects PC12 Cells against H_2_O_2_-Induced Cytotoxicity

**Figure 4.**
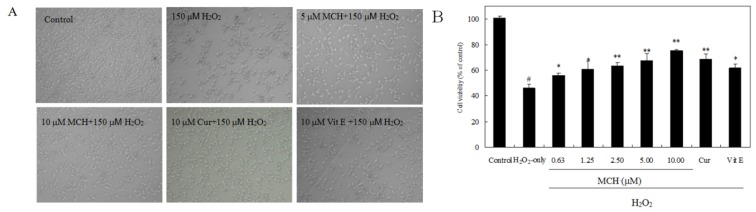
The protective effect of MCH on H_2_O_2_-induced damage in PC12 cells. (**A**) Cell morphology, Original magnification: ×100; (**B**) Cell viability was determined by the MTT reduction assay. Cells were pretreated with 0.63–10.00 μM MCH or 10.00 μM vitamin E (Vit E), 10.00 μM curcumin (Cur) for 30 min and then incubated in the presence of 150 μM H_2_O_2_ for 3 h. The control group was treated with 0.2% (*v*/*v*) DMSO under the same culture conditions. Data presented are the means ± SD of results from three independent experiments (^#^ *p* < 0.01 versus control; * *p* < 0.05 and ** *p* < 0.01 *versus* H_2_O_2_-treated cells).

Compared with normal PC12 cells, cells exposed to 150 μM H_2_O_2_ for 3 h exhibited morphological alteration, including a marked decrease in cell number, cell shrinkage and membrane blebbing (Figure 4A). The pretreatment of 5 μM MCH or 10 μM curcumin could mitigate such cell damages. As estimated by MTT assay, cell viability was markedly decreased to 46.2% after a 3 h exposure to 150 μM H_2_O_2_. However, when cells were pre-incubated with MCH (0.63–10.00 μM) for 30 min, cell toxicity was significantly attenuated in a dose-dependent manner (Figure 4B). Pretreatment of PC12 cells with MCH (0.63–10.00 μM) and 10 μM curcumin for 30 min significantly elevated the cell viability of PC12 cells to a range of 60.9%–75.4% and 72.7%, respectively. A 50% reduction in H_2_O_2_-induced cell death (*EC*_50_) was achieved with the reference substance curcumin at 9.85 ± 0.66 μM and the positive control vitamin E at 13.08 ± 0.71 μM, respectively. MCH possessed remarkable PC12 protective capacities, with *EC*_50_ values of 7.02 ± 0. 32 μM (data not shown).

**Figure 5.**
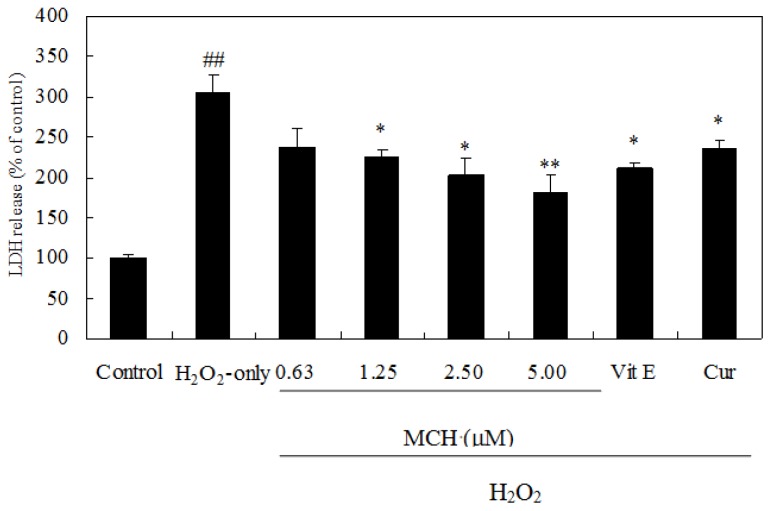
Inhibition of LDH release by MCH. Cells were pretreated with 0.63–5.00 μM MCH or 10.00 μM vitamin E (Vit E), 10 μM curcumin (Cur) for 30 min and then incubated in the presence of 150 μM H_2_O_2_ for 3 h. The control group was treated with 0.2% (*v*/*v*) DMSO under the same culture conditions. Data presented are the means ± SD of results from three independent experiments (^##^ *p* < 0.01 *versus* control; * *p* < 0.05 and ** *p* < 0.01 *versus* H_2_O_2_-treated cells).

To further investigate the protective effects of MCH, the release of LDH, another indicator of cell toxicity, was performed. As shown in Figure 5, a significant increase in LDH release was observed after 3-h exposure to 150 μM H_2_O_2_. MCH attenuated markedly this increase. Our results clearly indicated that H_2_O_2_-induced cytotoxicity in PC12 cells was attenuated in the presence of MCH.

### 2.5. MCH Reduced Lipid Peroxidation and Rescued Loss of Antioxidant Enzyme Activities in H_2_O_2_-Treated PC12 Cells

**Figure 6.**
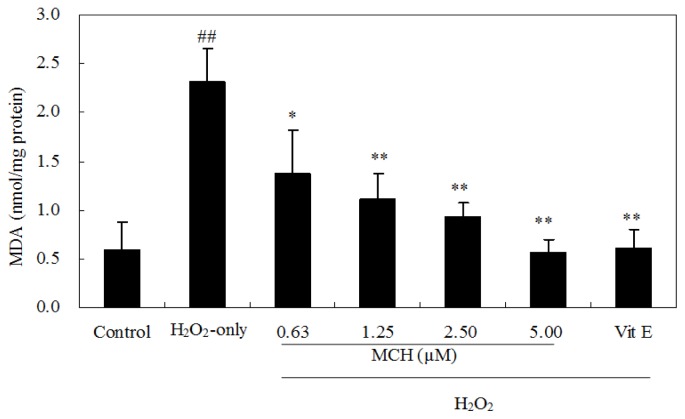
Effect of MCH on the MDA level in PC12 cells. Cells were pretreated with 0.63–5.00 μM MCH or 10 μM vitamin E (Vit E) for 30 min and then incubated in the presence of 150 μM H_2_O_2_ for 3 h. The control group was treated with 0.2% (*v*/*v*) DMSO under the same culture conditions. Data presented are the means ± SD of results from three independent experiments (^##^ *p* < 0.01 *versus* control; * *p* < 0.05 and ** *p* < 0.01 *versus* H_2_O_2_-treated cells).

**Figure 7.**
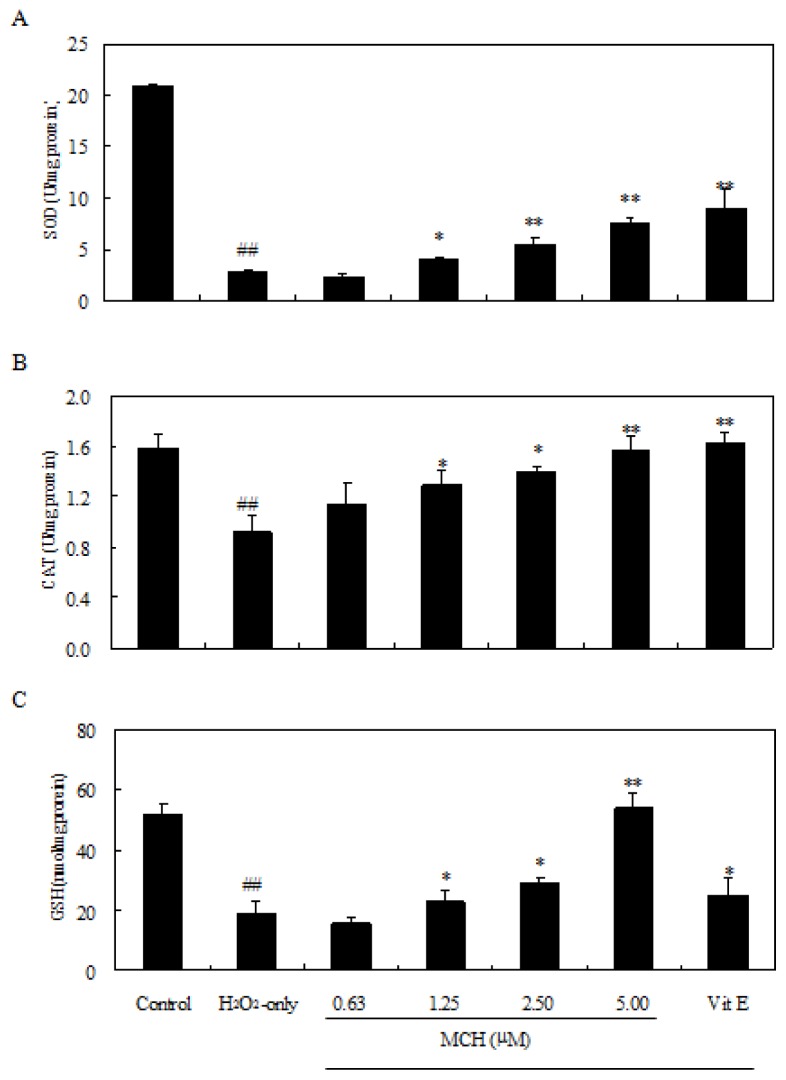
Effect of MCH on the activity of SOD (**A**); CAT (**B**) and intracellular GSH level (**C**) in PC12 cells. Cells were pretreated with 0.63–5.00 μM MCH or 10.00 μM vitamin E (Vit E) for 30 min and then incubated in the presence of 150 μM H_2_O_2_ for 3 h. The control group was treated with 0.2% (*v*/*v*) DMSO under the same culture conditions. Data presented are the means ± SD of results from three independent experiments (^##^ *p* < 0.01 *versus* control; * *p* < 0.05 and ** *p* < 0.01 *versus* H_2_O_2_-treated cells).

Treatment of PC12 cells with 150 μM H_2_O_2_ for 3 h caused an increase of the intracellular MDA level, while pre-incubation of cells with 0.63–5.00 μM MCH markedly attenuated this increase (Figure 6). Additionally, the exposure of PC12 cells to 150 μM H_2_O_2_ caused a decrease in the activity of SOD, CAT (Figure 7A,B), and GSH level (Figure 7C), respectively. Pretreatment with MCH significantly attenuated the decrease of GSH level and the activity of SOD and CAT in a dose-dependent manner.

### 2.6. MCH Prevents H_2_O_2_-Induced ROS Generation

To investigate whether MCH could prevent H_2_O_2_-induced ROS generation and resulting oxidative stress, we next measured the ROS production in the cells by using the fluorescence probe DCFH-DA. Exposure of the cells to 150 μM H_2_O_2_ for 3 h significantly increased the intracellular ROS level to 426.1% of the control (Table 2). PC12 cells pretreated with 0.63–5.00 μM MCH before H_2_O_2_ exposure markedly reduced the ROS levels in PC12.

### 2.7. MCH Protected PC12 Cells against H_2_O_2_-Induced Apoptosis

**Figure 8.**
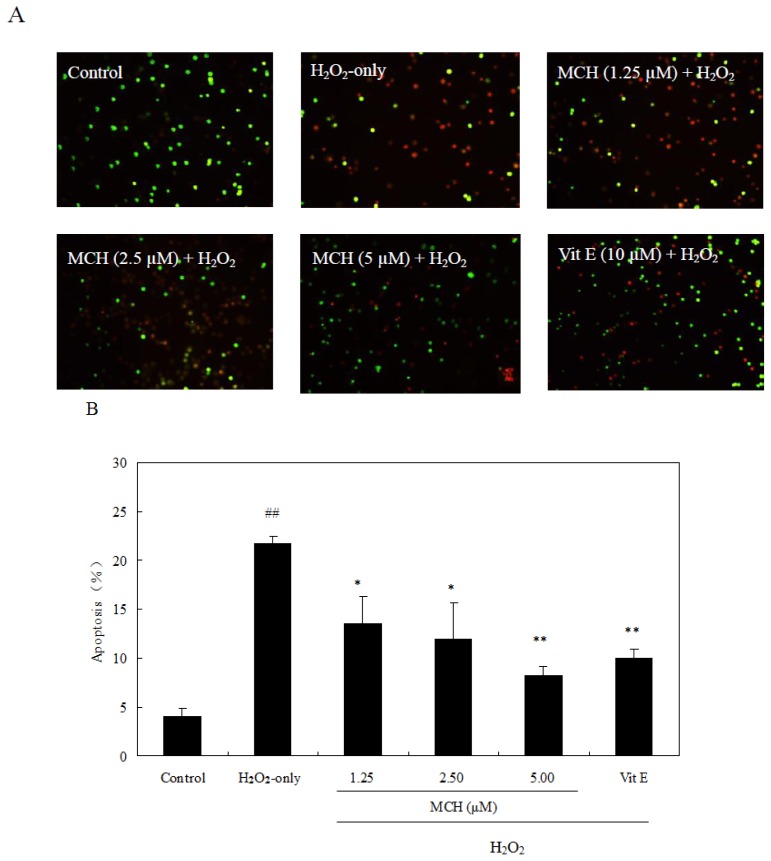
Inhibitory effect of MCH on H_2_O_2_-induced apoptosis in PC12 cells. (**A**) Morphological assessment by acridine orange-ethidium bromide (AO/EB) staining under the microscope (×100); (**B**) AV-FITC/PI analysis by flow cytometry method showed the percentage of apoptotic cells. Cells were pretreated with 0.63–5.00 μM MCH or 10.00 μM vitamin E (Vit E) for 30 min and then incubated in the presence of 150 μM H_2_O_2_ for 3 h. The control group was treated with 0.2% (*v*/*v*) DMSO under the same culture conditions. Data presented are the means ± SD of results from three independent experiments (^##^ *p* < 0.01 *versus* control; * *p* < 0.05 and ** *p* < 0.01 *versus* H_2_O_2_-treated cells).

To test whether H_2_O_2_-induced cell death via apoptosis, AO/EB staining was used. After 3 h treatment with 150 μM H_2_O_2_ alone, chromatin in most of PC12 cells was stained by orange, indicating that cells were apoptotic (Figure 8A). The treatment of MCH at 1.25–5.00 μM on H_2_O_2_-treated PC12 cells significantly reduced the number of stained cells. The percentage of apoptotic cells treated with 150 μM H_2_O_2_ was approximately 21.8% by the AV/PI assay (Figure 8B). MCH (1.25–5.00 μM) and vitamin E (10 μM) significantly attenuated cell apoptosis induced by H_2_O_2_ to 8.3%–13.5% and 10.0%, respectively.

### 2.8. MCH Prevented Loss of MMP in PC12 Cells

A decreasing MMP (*ψ*_m_) is associated with mitochondrial dysfunction linked to apoptosis [16]. Thus, we next evaluated the effect of MCH on MMP of PC12 cells with flow cytometry using the *ψ*_m_-dependent fluorescent rhodamine123. After incubation of PC12 cells with 150 μM H_2_O_2_ for 3 h, the MMP level was rapidly reduced to 48.32% ± 0.41% of the control (Table 2). Pretreatment with MCH at 0.63–5.00 μM or vitamin E at 10.00 μM significantly reduced the changes in MMP induced by H_2_O_2_. MCH increased the levels of MMP by 26.5%–33.7%, while vitamin E increased the level of MMP by 38.3%, as compared to the PC12 cells treated with H_2_O_2_, which indicated that MCH protected cells against the H_2_O_2_-induced lowering of MMP in PC12 cells.

### 2.9. MCH Inhibited H_2_O_2_-Induced Expression of Cleaved Caspase-3 in PC12 Cells

**Figure 9.**
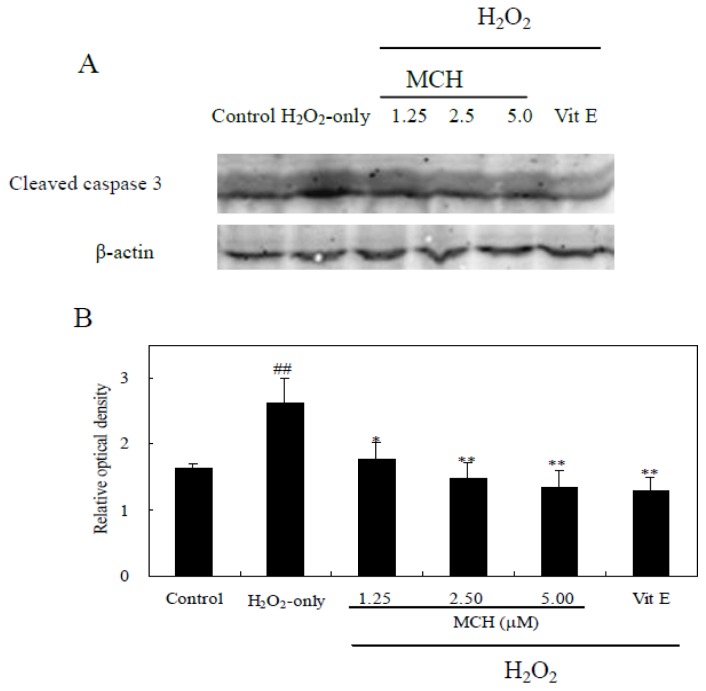
Effect of MCH on the expression of cleaved caspase-3 levels in PC12 cells. Cells were pretreated with MCH at 1.25–5.00 μM or 10.00 μM vitamin E (Vit E) for 30 min and then incubated in the presence of 150 μM H_2_O_2_ for 3 h. The control group was treated with 0.2% (*v*/*v*) DMSO under the same culture conditions. (**A**) The expression of cleaved caspase-3. β-actin was used for normalization and verification of protein loading; (**B**) Quantitative cleaved caspase-3 expression after normalization to β-actin. Data presented are the means ± SD of results from three independent experiments (^##^ *p* < 0.01 *versus* control; * *p* < 0.05 and ** *p* < 0.01 *versus* H_2_O_2_-treated cells).

Cleaved caspase-3 is the key apoptotic executive protein, which could be activated by both death-receptor and mitochondrial pathways [17]. Figure 9 showed that H_2_O_2_ treatment significantly up-regulated the expression of caspase-3, and this was markedly reduced by pretreatment with MCH (1.25–5.00 μM) or vitamin E (10 μM).

### 2.10. MCH Promoted the Expression of Nrf2 in PC12 Cells

**Figure 10.**
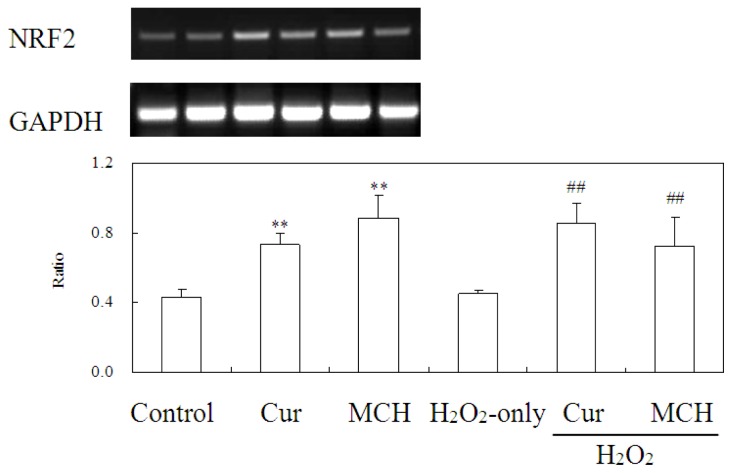
Effects of MCH on the expression of Nrf2 genes. PC12 cells were treated for 24 h with 150 μM H_2_O_2_ in the absence/presence of 5 μM of Cur/MCH pretreatment (30 min). The expression of Nrf2 was determined by semi-quantitative RT-PCR. GAPDH was used as an internal control in both experiments. Ratio, band densitometric of Nrf2 gene/band densitometric of GAPDH. (** *p* < 0.01 *versus* control and ^##^ *p* < 0.01 *versus* H_2_O_2_-treated cells).

Curcumin does not only have the ability to scavenge free radicals, but most importantly, it can strongly induce the expression of many antioxidant genes in mammalian cells including the activation of the transcription factor NF-E2-related factor 2 (Nrf2), a master regulator of intracellar detoxifying systems [18]. To determine the effects of MCH on Nrf2 gene transcription, Nrf2 mRNA expression was analyzed by semi-quantitative RT-PCR (Figure 10). The mRNA expression of Nrf2 was significantly induced by the treatment with curcumin or MCH at 5 μM. After incubation of PC12 cells with 150 μM H_2_O_2_ for 24 h, higher expression level of Nrf2 gene was retained with the pretreatment of curcumin or MCH at 5 μM, which was approximately 1.9, 1.6 fold (*versus* H_2_O_2_-only treatment), respectively.

## 3. Discussion

Curcumin, a natural phenolic diarylheptanoid was reported to have neuroprotective effects via reducing oxidative stress [3]. The potent chain-breaking antioxidant activity of curcumin has currently received remarkable interest for its typical radical trapping ability [19]. Although a lot of work has been reported on the potential use of curcumin as an antioxidant, the search for new derivatives or analogues is ongoing to develop compounds that have a better antioxidant activity [5]. In our present study, a novel curcumin analogue MCH, proved effective in superoxide anion scavenging and PC12 cell protection from oxidative damage. Although MCH was less potent than curcumin in scavenging capacity on DPPH and hydroxyl radical by chemical reaction *in vitro*, it exhibited significant protective effects on PC12 cells against oxidative damage with an *EC*_50_ value of 7.02 ± 0. 32 μM, similarly effective as curcumin (*EC*_50_ = 9.85 ± 0.66 μM). Moreover, MCH and curcumin exhibited a similar slight cytotoxic effect at concentrations greater than 30 μM (Figure 3). As the presence of the active methylene group and β-diketone moiety contributed to the instability of curcumin under physiological conditions [12], we adopted the chemical synthesis of MCH, a mono-carbonyl analogue of curcumin by eliminating the unstable methylene group and β-diketone moiety, which showed a much better stability in the test (Figure 2). The distinct protective ability against oxidative damage as well as the much improved stability makes the novel curcumin analogue MCH a promising antioxidant.

Cell culture of PC12 cells is commonly used as a screening model for testing the prevention of ROS-induced neuronal death [14]. H_2_O_2_ can generate detrimental hydroxyl radicals and increase the ROS levels in cells. In this study, treatment of PC12 cells with 150 μM H_2_O_2_ for 3 h caused significant increase of the intracellular ROS level (Table 2), elevation of oxidative stress characterized by MDA production (Figure 6) and LDH release (Figure 5). Although MCH exhibited weaker scavenging capacity on DPPH and hydroxyl radicals *in vitro* (Table 1), it markedly reduced the ROS levels in PC12 cells after the pretreatment at 0.63–5.00 μM. In addition to possible direct free radical scavenging, MCH may have indirect effects, such as the modulation of endogenous antioxidant enzymes to reduce ROS levels. Among the most important defenses against oxygen radicals are CAT and SOD enzymes. SOD catalyses dismutation of the superoxide anion into H_2_O_2_ and CAT detoxifies H_2_O_2_ to oxygen and water [20]. The combined action of these two enzymes reduces ROS levels in cells and repairs oxidized injury on membrane components. It was reported that SOD and CAT activities in PC12 cells were reduced after the treatment of H_2_O_2_ [21]. In this study, when PC12 cells were pre-incubated with MCH, a rescue on loss of SOD and CAT activities as well as cell survival was observed in H_2_O_2_-treated PC12 cells (Figures 4 and 7). On the other hand, GSH is another key regulator of intracellular redox potential. It serves as an electron donor to unstable ROS and performs cell-protective antioxidant role, cycling between its reduced form GSH and oxidized form glutathione disulfide (GSSG). Many studies have shown that GSH depletion is associated with ROS generation, mitochondrial dysfunction and apoptosis induction [22,23]. Our study showed that PC12 cells treated with H_2_O_2_ caused the decrease in GSH content by 62.6%, however, pretreatment of PC12 cells by MCH at 5 μM resulted in a 3.3-fold increase in GSH level compared to the H_2_O_2_-treated cells (Figure 7C). This result indicated that GSH metabolism could be regulated by MCH as another detoxifying system to prevent damage caused by ROS in PC12 cells.

NF-E2-related factor 2 (Nrf2), a cap “n” Collar (CNC) transcription factor, is a key regulator of an expansive set of antioxidant response element (ARE)-mediated gene expression to remove ROS [24]. Curcumin was found to have the capacity to upregulate Nrf2 expression and protect the rat brain from focal ischemia [18]. Bisdemethoxycurcumin is more active than curcumin in inducing Nrf2-mediated induction of heme oxygenase-1 (HO-1) [25]. A novel water soluble curcumin derivative (NCD) could induce HO-1 in the pancreatic and cardiac tissues of the diabetic rats and exhibit anti-diabetic activities [26]. The α,β-unsaturated diketone moiety in curcumin is a Michael reaction acceptor, which can activate Nrf2 followed by up-regulation of HO-1 expression and induce the phase-II response [27,28]. Jeong *et al*. (2006) also suggested that the presence of methoxy groups in the *ortho* position on the aromatic ring of curcuminoids was essential to enhance HO-1 expression. In our newly synthesized curcumin analog MCH, two Michael reaction acceptors (α,β-unsaturated diketone group) and methoxy groups in the ortho position on the aromatic ring were remained (Figure 1). We found that MCH and curcumin both significantly increased mRNA expression of Nrf2 after 30 min of incubation (Figure 10). Under the oxidative injury caused by H_2_O_2_, MCH upregulated cellular Nrf2 expression at the transcriptional level, suggesting a possible involvement of the Nrf2 pathway in MCH-induced cytoprotective effect in PC12 cells.

Excessive production of ROS causes oxidative damage to cellular proteins, lipids, nucleic acids and ultimately leads to an apoptotic or necrotic cell death pathway in several cell types [29]. In this study, treatment of PC12 cells with 150 μM H_2_O_2_ for 3 h caused a marked apoptosis and decrease in cell survival (Figures 4 and 8). MCH could attenuate cell apoptosis and reduce cell death. It was reported that the increase of ROS may impair mitochondrial function, leading to a disruption of MMP and the release of apoptosis-inducing factors, which activate the caspase cascade to apoptosis [30]. In the present study, treatment of PC12 cells with H_2_O_2_ caused the intracellular accumulation of ROS and further induced the loss of MMP (Table 2). The subsequent disruption of MMP caused an increase in cleaved caspase-3 activities and eventually apoptosis (Figure 9). However when PC12 cells were pretreated with MCH, the accumulation of ROS, the loss of MMP, the increase of cleaved caspase-3 activities and apoptosis were attenuated. These results suggested that ROS scavenging effects of MCH might be important in reducing the level of apoptosis and the cell death induced by H_2_O_2_.

## 4. Materials and Methods

### 4.1. Materials

The PC12 cell line was purchased from the Chinese Type Culture Collection (Shanghai Institute of Cell Biology, Chinese Academy of Science, Shanghai, China). Curcumin, 1,1-diphenyl-2-picrylhydrazyl (DPPH), rhodamine123 (Rh123), nicotinamide adenine dinucleotide-reduced (NADH), α-tocopherol, nitroblue tetrazolium (NBT) and phenazine methosulfate (PMS), 3-(4,5-dimethylthiazol-2-yl)-2,5-diphenyltetrazoliumbromide (MTT) and *N*-2-hydroxyethylpiperazine-*N*′-2-ethanesulfonic acid (HEPES) were purchased from Sigma-Aldrich (St. Louis, MO, USA). 2-Thiobarbituric acid (TBA), trichloroacetic acid (TCA), hydrogen peroxide (H_2_O_2_), and ascorbic acid from Sinopharm Chemical Reagent Company (Shanghai, China). RPMI 1640 medium was purchased from Gibco (Carlsbad, CA, USA). Calf serum was purchased from Hangzhou Sijiqing Co., Ltd. (Hangzhou, China). The kits for lactate dehydrogenase (LDH) activity, GSH activity, malondialdehyde (MDA) activity, SOD activity and ROS assay were purchased from Beyotime Institute of Biotechnology (Shanghai, China). Annexin V (AV)-FITC/propidium iodide (PI) kit was purchased from KeyGEN (Nanjing, China). The cleaved caspase-3 and β-actin antibody was purchased from Sangon Biotech Co., Ltd. (Shanghai, China). All other chemicals were analytical reagent (AR)-grade.

### 4.2. Synthesis of MCH

MCH was synthesized according to Figure 1. Cyclohexanone (1.1 g, 0.011 mol) and 3,5-dimethoxybenzaldehyde (3.6 g, 0.022 mol) was added into 10 mL of 10% NaOH in ethanol solution, and to make sure the reaction was complete; the solution was stirred overnight at room temperature. Ethanol was removed *in vacuo* and ethyl acetate (20 mL × 3) was added to extract, the organic potions was washed by H_2_O and brine, and dried by anhydrous sodium sulfate. The crude product was obtained by removing solution *in vacuo*, which was purified by silica chromatography column (petroleum ether:ethyl acetate = 15:1 (*v*/*v*)), to obtain the product as pale yellow needle-like crystals 3.55 g, yield 81.8%, m.p.: 135.7~136.5 °C. ^1^H-NMR (400 MHz, CDCl_3_), δ (ppm): 7.71 (s, 2H, =CH), 6.60 (d, 4H, *J* = 2.1 Hz, ArH), 6.46 (t, 2H, *J* = 2.1 Hz, ArH), 3.82 (s, 12H, OCH_3_), 2.92 (t, 4H, *J* = 5.5 Hz, CH_2_), 1.78 (quint, 2H, *J* = 6.6 Hz, CH_2_). ^13^C-NMR (400 MHz, CDCl_3_), δ (ppm): 190.525, 160.833, 137.995, 137.243, 136.866, 108.592, 101.041, 55.702, 28.813, 23.181. HRMS (AP-ESI) *m*/*z* Calcd. For C_24_H_26_O_5_ [M + H]^+^ 395.1853, Found 395.1869.

### 4.3. DPPH Radical Scavenging Assay

DPPH radical scavenging activity was measured as described in our previous work [31]. α-Tocopherol and vitamin C were used as positive control and the sample solution without DPPH was used as sample blank.

### 4.4. Superoxide Radical Scavenging Assay

The superoxide radical scavenging ability was measured as described in our pervious work [32]. The color reaction of superoxide radicals and nitroblue tetrazolium (NBT) was detected at 560 nm using a microplate reader (Molecular Devices, Sunnyvale, CA, USA). Vitamin C was used as the positive control in this experiment.

### 4.5. Hydroxyl Radical Scavenging Assay

The hydroxyl radical scavenging activity was measured using the Fenton reaction assay as described in our previous work [31]. Curcumin was used as a reference compound.

### 4.6. Stability Studies *in Vitro*

The stability of the tested compounds was measured according to the procedure of Fang *et al*. [33] with some modification. MCH and curcumin were dissolved in 100% DMSO at 5 mM for the measurement. When measurement began, 80 μL of the tested compound solution was diluted with 4 mL of 1.5 mM phosphate-buffered solution (pH 7.4). The degradation process was followed by visible absorption spectroscopy at 410 nm in a rectangular quartz cuvette with a 1 cm optical path length at 37 °C on a Shimadzu 2600 UV/visible spectrophotometer in the presence or absence of 0.1% FBS. The absorption of the solution measured at 0 min was recorded as *A*_0_; the absorption of the solution measured at other time was recorded as *A_t_*. All results are representative of three independent experiments.

### 4.7. Culture of PC12 Cells

PC12 cells were cultured in RPMI 1640 medium (pH 7.4) with 10% calf serum at 37 °C under 5% CO_2_. Before treatment, cells were plated at appropriate density on 96- or 6-well culture plates and cultured for 24 h. In all experiments, cells were pretreated for 30 min with indicated concentrations of MCH or vitamin E for 30 min, and later, 150 μM H_2_O_2_ was added to the medium for 3 h. MCH was not removed after the addition of H_2_O_2_. MCH was freshly prepared as stock solution in dimethylsulfoxide (DMSO) and diluted with RPMI 1640 medium before every experiment. DMSO (0.2%, *v*/*v*) had no protective or toxic effect by itself. The control group was performed in the presence of 0.2% (*v*/*v*) DMSO under the same culture conditions.

### 4.8. Measurement of Cell Viability

To investigate the cytotoxicity of MCH and curcumin in PC12 cells, cells (6.0 × 10^3^ per well) were seeded into 96-well plates and exposed to various concentration of MCH and curcumin for 20 h. 10 μL MTT at 5 mg/mL was added to each well. The cells were then incubated at 37 °C for another 4 h and then 10% sodium dodecyl sulfate (SDS) in 0.01 M HCl was added to each well. The absorbance was detected at 570 nm with a microplate reader KLx808 (Bio-Tek, Norcross, GA, USA). Cell viability is expressed as a percentage of untreated cells.

To investigate the protective effect of MCH on H_2_O_2_-induced damage in PC12 cells, cells were pretreated with 0.63–5.00 μM MCH or 10 μM Vit E, 10 μM curcumin for 30 min and the control group was treated with 0.2% (*v*/*v*) DMSO under the same culture conditions. Then cells were incubated in the presence of 150 μM H_2_O_2_ for 3 h. The protection of the compound on H_2_O_2_-induced damage in PC12 cells was expressed as an *EC*_50_, defined as the concentration required for 50% reduction in H_2_O_2_-induced cell death compared with control cells and determined from at least three independent experiments.

### 4.9. Lactate Dehydrogenase (LDH) Assay

PC12 cells in 96-well plates were cultured and treated according to the procedures described above. After the treatment, the medium were harvested for the spectrophotometrical determination of the amount of LDH released by cells using an assay kit (Nanjing Jiancheng Bioengineering Institute, Nanjing, China) according to the protocol of the manufacturer, and the absorbance of the samples was read at 490 nm.

### 4.10. Measurement of Intracellular ROS Accumulation

The production of intracellular ROS was quantified using a DCFH-DA assay [34]. PC12 cells were loaded with 10 μM DCFH-DA and incubated at 37 °C for 30 min. And then washed three times with PBS (0.1 M, pH 7.4) and treated with MCH for 30 min, followed by the addition of 150 μM H_2_O_2_. After 3 h of incubation, the cells were harvested and suspended in PBS. The fluorescence intensity was measured by a flow cytometer Beckman-Coulter FC500 (Brea, CA, USA) at an excitation wavelength of 488 nm and an emission wavelength of 530 nm.

### 4.11. Assays for GSH Content and Antioxidant Enzymes

To determine GSH level and the activity of CAT and SOD, PC12 cells (5 × 10^5^ cells/mL) were plated in culture plates and cultured for 24 h. After the treatments, cells were washed twice in ice-cold PBS and homogenized. The homogenate was centrifuged at 10,000 rpm for 10 min at 4–8 °C. The supernatants were collected for the assay. The catalase activity was assessed according to the method described by Cohen *et al.* [35]. The activities of SOD and the content of GSH were all determined by using assay kits (Beyotime Institute of Biotechnology, Haimen, China). Protein content was measured by the Bradford method using bovine serum albumin as standard [36].

### 4.12. Measurement of Mitochondrial Membrane Potential (MMP)

Alteration in MMP was analyzed by flow cytometry using the Rh123 fluorescent dye as previously described [37]. Briefly, cells were resuspended in PBS (0.1 M, pH 7.4), incubated with Rh123 (10 mM) at 37 °C for 30 min, washed twice with PBS (0.1 M, pH 7.4). The cellular fluorescence intensity was quantified using flow cytometry Beckman-Coulter FC500 (Brea, CA, USA) at an excitation wave-length of 480 nm and an emission wavelength of 530 nm. Cellular mitochondrial membrane potential was expressed as a percentage of control cells.

### 4.13. Apoptosis Analysis by AO/EB and Annexin V-FITC/PI Staining

Apoptotic morphology was investigated by AO and EB staining [31], cells were harvested and washed twice with PBS (0.1 M, pH 7.4) after the treatment and then stained with 100 μg/mL AO and EB for five min. Nuclei were visualized and photographed under a fluorescent microscope Olympus CKX41 (Tokyo, Japan).

Cell Apoptosis was also observed under annexin V-FITC/PI Staining. The treated cells were washed twice with ice-cold PBS (0.1 M, pH 7.4). Cells were resuspended in 500 μL of 1× binding buffer, 5 μL annexin V-FITC added, 5 μL PI, and then incubated at room temperature for 15 min in the dark. The cells were analyzed by a flow cytometry Beckman-Coulter FC500 (Brea, CA, USA).

### 4.14. Analysis of Cleaved Caspase-3 Expression with Western Blot

Cells were harvested, washed in PBS (0.1 M, pH 7.4), centrifuged, and resuspended in cell lysis solution containing 20 mM Tris (pH 7.5), 150 mM NaCl, 1% Triton X-100 and several protein inhibitors such as sodium pyrophosphate, β-glycerophosphate, EDTA, Na_3_PO_4_ and leupeptin (Beyotime Institute of Biotechnology, Haimen, China). The protein concentration of each extract was determined by the bicinchoninic acid (BCA) assay [38]. Western blot was performed according to the procedure of Towbin *et al*. [39] with some modification. Cell extracts (60 μg protein/lane) were separated by electrophoresis on 12% SDS-polyacrylamide gels. Proteins were subsequently transferred to a nitrocellulose membrane, which was then incubated with 5% skimmed milk in Tris-buffered saline with 0.1% Tween 20 (TBST) for 1 h at room temperature. Afterward, the membranes were incubated with the primary antibodies (rabbit monoclonal anti-cleaved caspase-3 (1:100) and anti-β-actin (1:100)) overnight at 4 °C. After washing with TBST, membranes were then incubated with FITC-labeled secondary antibodies (Beyotime Institute of Biotechnology, Haimen, China), and the signal was read with an Odyssey^®^ Western Blot Analysis system (Li-COR Biosciences, Lincoln, NE, USA). The signal intensity of primary antibody binding was quantitatively analyzed with Sigma Scan Pro 5 (Systat Software Inc., San Jose, CA, USA)

### 4.15. Semi-Quantitative RT-PCR Analysis

RT-PCR was used to analyze the level of Nrf2 mRNA. Total RNA was isolated from 5 × 10^6^ treated PC12 cells at logarithmic phase by using RNAiso Plus (Takara, Shiga, Japan) according to the manufacturer’s instructions. Forward and reverse primers were 5′-CCATTTACGGAGACCCAC-3′ and 3′-CTTATTTCAACGGCGAGT-5′ for Nrf2, 5′-AAATGGGTGATGCTGGTG-3′ and 3′-TGAGCGAGTTCTAACAGTCG-5′ for GAPDH [40]. Reverse transcription used reagents from Promega following the manufacturer’s instructions. The RT-PCR products were separated on 1.2% agarose gel and the intensity of each band was quantified using SynGene software (SynGene, Cambridge, UK) and expressed in arbitrary units (GeneGenius Super 12, Syngene, Cambridge, UK).

### 4.16. Statistical Analysis

The experimental date was expressed as means ± standard deviations. One-way analysis of variance (ANOVA) was carried out to determine significant differences between the means by SPSS (version 11.0, SPSS Inc. Chicago, IL, USA).

## 5. Conclusions

In summary, MCH, a mono-carbonyl analogue of curcumin was synthesized simply and effectively. MCH exhibited better protective ability against H_2_O_2_-induced oxidative damage in PC12 cells than curcumin and furthermore, the *in vitro* stability of MCH is also improved compared with curcumin. The protective effect was, at least in part, attributed to its scavenging activity on superoxide anion, the prevention of GSH loss, the ability of modulating endogenous antioxidant enzymes and the possible involvement of the Nrf2 pathway. We propose that MCH attenuates H_2_O_2_-induced apoptosis through direct and indirect scavenging of ROS, leading to inhibition of the mitochondria-mediated apoptotic pathway. Our results suggest the potential for MCH to be used in treating diseases in which free radical and oxidative damage are involved.

## Figures and Tables

**Table 1.** Free radical scavenging activities and reducing power of MCH (a).

Compound	*EC*_50_ (μM) (b)		

	DPPH	Superoxide anion	Hydroxyl radical
MCH	466.79 ± 30.50 ^**^	64.78 ± 7.72 ^**^	94.03 ± 1.12 ^##^
Curcumin	14.24 ± 0.60	88.09 ± 5.97 ^**^	58.84 ± 3.14
Vitamin C	13.00 ± 0.37	100.93 ± 4.10	n.d. (c)

(a) Data are expressed as the mean ± SD, *n* = 3; (^**^ *p* < 0.01 MCH or curcumin versus vitamin; ^##^ *p* < 0.01 MCH *versus* curcumin);

(b) *EC*_50_: the effective concentration at which the antioxidant activity was 50%; 1,1-diphenyl-2-picrylhydrazyl (DPPH), superoxide anion or hydroxyl radicals were scavenged by 50%;

(c) n.d. = not detected.

**Table 2.** Effects of MCH on the changes of the MMP and ROS level in PC12 Cells (a).

Treatment	ROS (percentage of the control %)	MMP (percentage of the control %)
Control (0.2% DMSO)	100 ± 7.35	100 ± 7.58
H_2_O_2_-only (150 μM)	426.09 ± 36.20 ^##^	48.32 ± 1.14 ^##^
MCH (0.63 μM) + H_2_O_2_	294.78 ± 57.32 ^**^	74.84 ± 5.05 ^**^
MCH (1.25 μM) + H_2_O_2_	269.18 ± 1.17 ^**^	74.01 ± 4.72 ^**^
MCH (2.50 μM) + H_2_O_2_	248.70 ± 22.55 ^**^	79.78 ± 1.55 ^**^
MCH (5.00 μM) + H_2_O_2_	231.59 ± 12.90 ^**^	82.01 ± 5.53 ^**^
Vit E (10.00 μM) + H_2_O_2_	241.16 ± 4.06 ^**^	86.64 ± 3.95 ^**^

(a) Cells were pretreated with 0.63–5.00 μM MCH or 10.00 μM vitamin E (Vit E) for 30 min and then incubated in the presence of 150 μM H_2_O_2_ for 3 h. The control group was treated with 0.2% (*v*/*v*) DMSO under the same culture conditions. Data presented are the means ± SD of results from three independent experiments (^##^ *p* < 0.01 *versus* control; ^**^ *p* < 0.01 *versus* H_2_O_2_-treated cells).
